# Correction: Bed-side measures for diagnosis of low muscle mass, sarcopenia, obesity, and sarcopenic obesity in patients with chronic kidney disease under non-dialysis-dependent, dialysis dependent and kidney transplant therapy

**DOI:** 10.1371/journal.pone.0250186

**Published:** 2021-04-08

**Authors:** Natália Tomborelli Bellafronte, Gabriel Ruiz Sizoto, Lorena Vega-Piris, Guillermina Barril Cuadrado, Paula Garcia Chiarello

The authors are listed out of order. The last author should be Paula Garcia Chiarello. Please view the correct author order, affiliations, and citation here:

Natália Tomborelli Bellafronte^1^, Gabriel Ruiz Sizoto^2^, Lorena Vega-Piris^3^, Guillermina Barril Cuadrado^4^, Paula Garcia Chiarello^5^

**1** Post-graduate Program in Health Sciences, Ribeirão Preto Faculty of Medicine, University of São Paulo, Ribeirão Preto, São Paulo, Brazil, **2** Nutrition and Metabolism Undergraduate Course, Ribeirão Preto Faculty of Medicine, University of São Paulo, Ribeirão Preto, São Paulo, Brazil, **3** Methodology Unit, Instituto de Investigación Sanitaria del Hospital Universitario de la Princesa, Madrid, Spain, **4** Nephrology Department, Hospital Universitario La Princesa, Madrid, Spain, **5** Department of Health Sciences, Ribeirão Preto Faculty of Medicine, University of São Paulo, Ribeirão Preto, São Paulo, Brazil

Bellafronte NT, Sizoto GR, Vega-Piris L, Cuadrado GB, Chiarello PG (2020) Bed-side measures for diagnosis of low muscle mass, sarcopenia, obesity, and sarcopenic obesity in patients with chronic kidney disease under non-dialysis-dependent, dialysis dependent and kidney transplant therapy. PLoS ONE 15(11): e0242671. https://doi.org/10.1371/journal.pone.0242671
