# Supplementary material for: Bed-side measures for diagnosis of low muscle mass, sarcopenia, obesity, and sarcopenic obesity in patients with chronic kidney disease under non-dialysis-dependent, dialysis dependent and kidney transplant therapy
Source: PLoS One. 2020 Nov 20;15(11):e0242671. doi: 10.1371/journal.pone.0242671 (PMC7679152; doi:10.1371/journal.pone.0242671)
Supplement: S1 Fig — (DOCX) [file pone.0242671.s001.docx]

86 Non SOB

82 Non SOB

4 SOB♦◊

2 SOB

2 SOB

67 Non OB

64 Non OB

3 OB●◊

20 OB

18 OB

2 Non OB

81 Non SCP

75 Non SCP

6 SCP▲■

3 SCP

3 Non SCP□

6 SCP

79 Normal HGS

70 Normal HGS

9 Low HGS*■

8 Low HGS

5 Low HGS

3 Normal HGS□

41 Low

MM

46 Normal MM

37 Normal MM

9 Low MM*▲●♦

39 Low MM

2 Normal MM

**S1 Figure**. Follow up graphic from first to second assessment of patients with participation in both assessment. HD, hemodialysis; HGS, hand grip strength; KTx, kidney transplant; MM, muscle mass; NDD, non dialysis-dependent; OB, obesity; PD, peritoneal dialysis; SCP, sarcopenic; SOB, sarcopenic obesity. *: 1 patient in common; ▲: 2 patient in common; ■: 5 patient in common; ●: 1 patient in common; □: 3 patients in common; ♦: 3 patients in common; ◊: 1 patient in common

***2^nd^ assessment***

**Chronic Kidney Disease Groups Treatment: follow up graphic of patients with participation in the first and second assessment**

**Body composition diagnostics: follow up graphic of patients with participation in the first and second assessment**

***1^st^ assessment***

23 KTx

15 PD

18 HD

25 NDD

***1^st^ assessment***

23 KTx

2 KTx

11 PD

2 HD

1 KTx

17 HD

1 PD

24 NDD

***2^nd^ assessment***
