# Supplementary material for: Bed-side measures for diagnosis of low muscle mass, sarcopenia, obesity, and sarcopenic obesity in patients with chronic kidney disease under non-dialysis-dependent, dialysis dependent and kidney transplant therapy
Source: PLoS One. 2020 Nov 20;15(11):e0242671. doi: 10.1371/journal.pone.0242671 (PMC7679152; doi:10.1371/journal.pone.0242671)
Supplement: S3 Fig — (DOCX) [file pone.0242671.s003.docx]

| A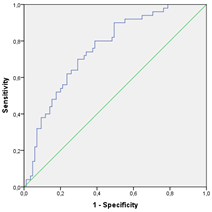 | B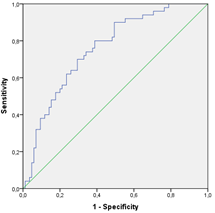 | C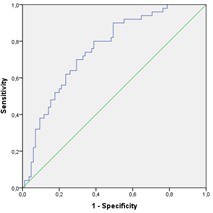 | D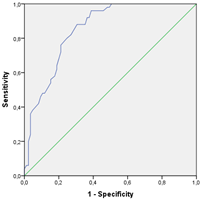 | E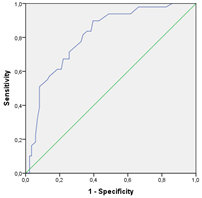 |
| --- | --- | --- | --- | --- |
| F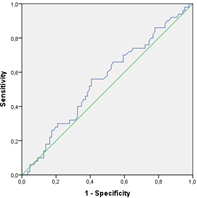 | G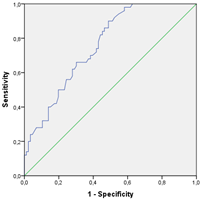 | H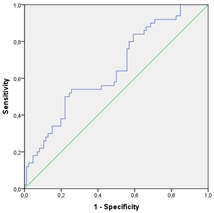 | I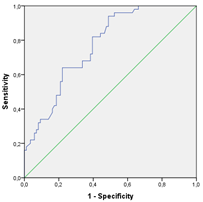 | J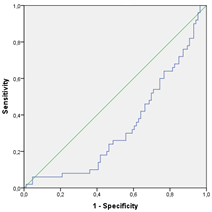 |
| K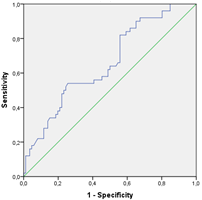 | L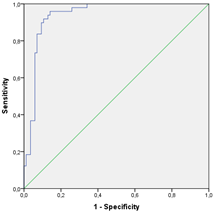 | M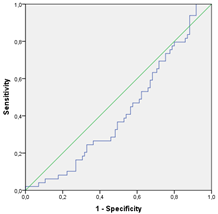 | N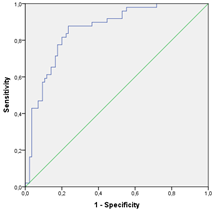 | O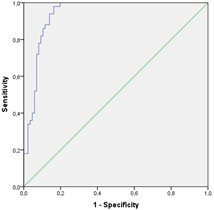 |
| **S3 Figure**. Receiver operation characteristic curve for low muscle mass diagnostic in male sample. (A) mid-arm muscle circumference; (B) arm muscle area; (C) corrected arm muscle area; (D) calf circumference; (E) adductor pollicis muscle thickness; (F) phase angle; (G) body cell mass (kg); (H) body cell mass index (kg/m^2^); (I) fat free mass (kg) by body composition monitor; (J) fat free mass (%) by body composition monitor; (K) fat free mass index (kg/m^2^) by body composition monitor; (L) predicted fat free mass (kg); (M) predicted fat free mass (%); (N) predicted fat free mass index (kg/m^2^); (O) appendicular fat free mass. From F to H, measures by bioelectrical impedance. From I to K, data generated by body composition monitor. From L to N, data predicted by Bellafronte equation equation [21] and O from Sergi equation [20]. Low muscle mass diagnostic as ALM<20kg assessed by dual energy X-ray absorptiometry [10]. | | | | |
