# Supplementary material for: Bed-side measures for diagnosis of low muscle mass, sarcopenia, obesity, and sarcopenic obesity in patients with chronic kidney disease under non-dialysis-dependent, dialysis dependent and kidney transplant therapy
Source: PLoS One. 2020 Nov 20;15(11):e0242671. doi: 10.1371/journal.pone.0242671 (PMC7679152; doi:10.1371/journal.pone.0242671)
Supplement: S4 Fig — (DOCX) [file pone.0242671.s004.docx]

| A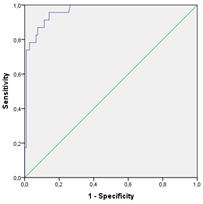 | B 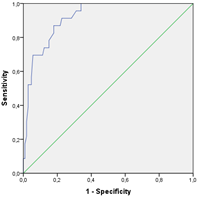 | C 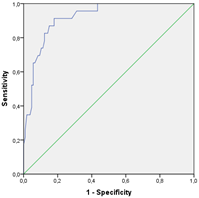 | D 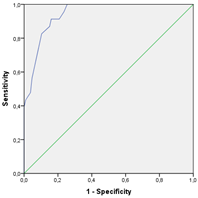 | | E 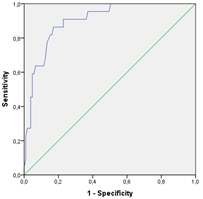 |
| --- | --- | --- | --- | --- | --- |
| F 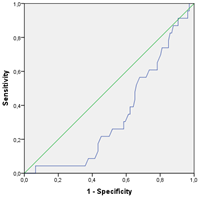 | G 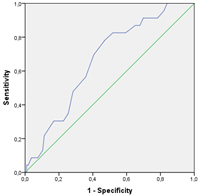 | H 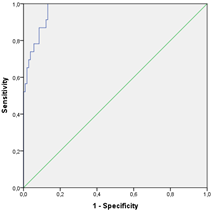 | I 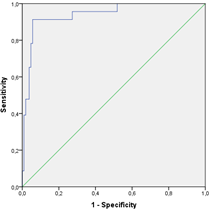 | | J 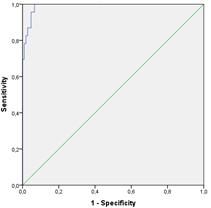 |
| K 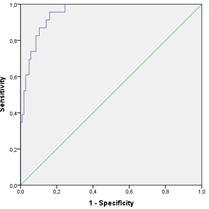 | L 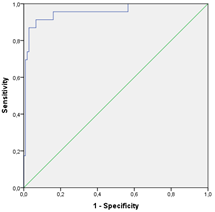 | M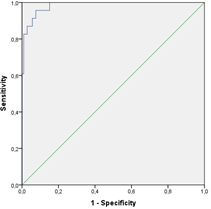 | | **S4 Figure**. Receiver operation characteristic curve for obesity diagnostic in female sample. (A) body mass index; (B) mid-arm circumference; (C) waist circumference; (D) waits circumference corrected by height; (E) triciptal skinfold thickness; (F) a body shape index; (G) conicity index; (H) fat mass (kg) by body composition monitor; (I) fat mass (%) by body composition monitor; (J) fat mass index (kg/m^2^) by body composition monitor; (K) predicted fat mass (kg); (L) predicted fat mass (%); (M) predicted fat mass index (kg/m^2^). From H to M, measures by bioelectrical impedance. From H to J, data generated by body composition monitor. From K to M, data predicted by Bellafronte equation [21]. Obesity diagnostic as FMI>13kg/m^2^ assessed by dual energy X-ray absorptiometry [14]. | |
