# Supplementary material for: Bed-side measures for diagnosis of low muscle mass, sarcopenia, obesity, and sarcopenic obesity in patients with chronic kidney disease under non-dialysis-dependent, dialysis dependent and kidney transplant therapy
Source: PLoS One. 2020 Nov 20;15(11):e0242671. doi: 10.1371/journal.pone.0242671 (PMC7679152; doi:10.1371/journal.pone.0242671)
Supplement: S1 Table — (DOCX) [file pone.0242671.s006.docx]

| **S1 TABLE. Clinical, anthropometric and body composition changes (second – first assessment) stratified by sex and official diagnostic** | | | | | | | | | | |
| --- | --- | --- | --- | --- | --- | --- | --- | --- | --- | --- |
| **Variables** | **Total prospective sample** | | **Pre-Sarcopenia** | | **Low Muscle Mass** | | **Obesity** | | **Sarcopenia** | |
|  | **Women**  **N = 40** | **Men**  **N = 46** | **Present**  **n = 8** | **Absent**  **n = 78** | **Present**  **n = 41** | **Absent**  **n = 46** | **Present**  **n = 20** | **Absent**  **n = 67** | **Present**  **n = 6** | **Absent**  **n = 43** |
|  | **±SD** | **±SD** | **±SD** | **±SD** | **±SD** | **±SD** | **±SD** | **±SD** | **±SD** | **±SD** |
| eGFR(ml/min/1.73m^2^) | 0.84±23.51 | -3.73±10.41 | -3.66±4.17 | -2.02±16.79 | -3.56±8.99 | -1.46±18.71 | -2.19±7.59 | -2.17±18.61 | -4.45±4.35 | -1.50±18.99 |
| KT/V | 0.31±0.85 | -0.09±0.30 |  | 0.17±0.72 | 0.28±0.81 | -0.13±0.28 | -0.12±0.37 | 0.19±0.74 |  | -0.13±0.28 |
| Weight (kg) | -1.33±3.27 | -0.56±3.32 | -0.73±2.09 | -0.99±3.39 | -0.79±3.08 | -1.03±3.52 | -0.78±3.67 | -0.95±3.21 | -0.23±2.18 | -1.08±3.56 |
| BMI (kg/m^2^) | -0.66±1.39 | -0.20±1.13 | -0.33±0.75 | -0.44±1.32 | -0.41±1.24 | -0.42±1.32 | -0.42±1.26 | -0.41±1.29 | -0.28±0.66 | -0.43±1.34 |
| HGS (kg) | -0.90±3.81 | -2.54±5.15 | **1.38±3.28*** | **-2.09±4.63*** | **-0.42±3.83*** | **-3.02±4.97*** | -3.12±6.05 | -1.38±4.09 | **2.30±3.27*** | **-3.09±5.07*** |
| Phase angle (°) | -0.07±0.51 | 0.00±0.57 | 0.12±0.55 | -0.04±0.55 | -0.04±0.51 | -0.02±0.57 | -0.08±0.67 | -0.02±0.50 | 0.36±0.37 | 0.00±0.58 |
| OH (L) | -0.01±1.00 | -0.24±1.67 | -0.26±1.01 | -0.15±1.42 | -0.07±1.00 | -0.19±1.69 | -0.11±1.75 | -0.14±1.29 | -0.60±0.84 | -0.29±1.68 |
| ALM (kg) | -0.75±1.59 | -0.75±1.65 | -0.61±1.00 | -0.81±1.63 | **-0.27±1.28*** | **-1.19±1.77*** | **-1.53±1.72*** | **-0.53±1.53*** | -0.36±0.93 | -1.27±1.72 |
| LM (kg) | -1.68±3.52 | -1.44±3.18 | -1.34±2.55 | -1.64±3.36 | **-0.58±2.46*** | **-2.43±3.76*** | **-3.03±3.26*** | **-1.13±3.24*** | -0.82±2.23 | -2.57±3.70 |
| TrFM (kg) | 0.14±1.75 | 0.48±2.44 | 0.35±1.19 | 0.34±2.23 | **-0.29±1.75*** | **0.88±2.32*** | **1.41±2.50*** | **0.02±1.93*** | 0.47±1.03 | 0.97±2.36 |
| FM (kg) | 0.33±3.12 | 0.82±4.02 | 0.29±2.16 | 0.66±3.76 | **-0.30±3.17*** | **1.41±3.84*** | **2.35±3.96*** | **0.09±3.38*** | 0.24±1.44 | 1.53±3.87 |
| ALMI (kg/m^2^) | -0.28±0.65 | -0.24±0.59 | -0.25±0.35 | -0.27±0.63 | **-0.09±0.51*** | **-0.41±0.67*** | **-0.54±0.63*** | **-0.18±0.60*** | -0.17±0.32 | -0.44±0.66 |
| LMI (kg/m^2^) | -0.64±1.45 | -0.45±1.15 | -0.57±0.91 | -0.56±1.33 | **-0.19±0.99*** | **-0.85±1.46*** | **-1.08±1.22*** | **-0.39±1.28*** | -0.40±0.79 | -0.89±1.46 |
| FMI (kg/m^2^) | 0.16±1.35 | 0.34±1.43 | 0.19±0.99 | 0.27±1.43 | **-0.09±1.33*** | **0.57±1.38*** | **0.90±1.41*** | **0.07±1.33*** | 0.14±0.69 | 0.61±1.37 |
| *: unpaired Student t-test between present and absent subgroups for the same diagnostic, p≤0.05. All diagnostic were applied in the first assessment. Cutoffs applied for Pre-sarcopenia diagnostic: for female, HGS<16kg; for male, HGS<27kg [10]. Cutoffs applied for low muscle mass diagnostic: for female, ALM<15kg; for male, ALM<20kg [10]. Cutoffs applied for obesity diagnostic: for female, FMI>13kg/m^2^; for male, FMI>9kg/m^2^[14]. For sarcopenia diagnostic, presence of diagnostic was applied if there is a concomitant presence of pre-Sarcopenia and low muscle mass diagnostics [10], and absence of sarcopenia diagnostic in the absence of both, pre-Sarcopenia and low muscle mass diagnostics. Sarcopenic obesity diagnostic was not evaluated as we had only 2 patients diagnosed in the first assessment and reevaluated. Data present in this table is referent to clinical, anthropometric and body composition changes (second – first assessment). ALM, appendicular lean mass; ALMI, appendicular lean mass index; BMI, body mass index; eGFR, estimated glomerular filtration rate; FM, fat mass; FMI, fat mass index; HGS, hand grip strength; LM, lean mass; LMI, lean mass index; OH, overhydration; TrFM, trunk fat mass. ALM, ALMI, FM, FMI and TrFM data by dual energy X-ray absorptiometry analysis. PhA and OH data by bioelectrical impedance analysis. | | | | | | | | | | |
