# Supplementary material for: Bed-side measures for diagnosis of low muscle mass, sarcopenia, obesity, and sarcopenic obesity in patients with chronic kidney disease under non-dialysis-dependent, dialysis dependent and kidney transplant therapy
Source: PLoS One. 2020 Nov 20;15(11):e0242671. doi: 10.1371/journal.pone.0242671 (PMC7679152; doi:10.1371/journal.pone.0242671)
Supplement: S2 Table — (DOCX) [file pone.0242671.s007.docx]

| **S2 TABLE. Clinical, anthropometric and body composition data from cross-sectional assessment of CKD patients stratified according to participation in the second assessment** | | | | |
| --- | --- | --- | --- | --- |
| **Variables** | **Categories** | **Group 1** | **Group 2** | **p** |
|  |  | **±SD** | **±SD** |  |
| n | **-** | 178 | 87 |  |
| Age (years) | **-** | 48±10 | 46±10 | 0.09 |
| Sex % (n) | **Male** | 50 (90) | 54 (47) | 0.78 |
| Ethnicity | **White** | 73 (130) | 74 (64) | 0.90 |
| Diabetes Mellitus % (n) | **Present** | 26 (47) | 27 (24) | 0.65 |
| Systemic Arterial Hypertension % (n) | **Present** | 65 (116) | 74 (64) | 0.55 |
| Dyslipidemia % (n) | **Present** | 16 (28) | 31 (27) | 0.07 |
| Dialysis or KTx time (month) | **-** | 72±52 | 80±79 | 0.51 |
| eGFR(ml/min/1.73m^2^) | **-** | 43.17±30.33 | 45.78±28.15 | 0.59 |
| KT/V | **-** | 1.80±0.70 | 1.98±0.59 | 0.21 |
| Weight (kg) | **-** | 72±15 | 71±17 | 0.74 |
| Body mass index (kg/m^2^) | **-** | 27±5 | 27±5 | 0.98 |
| Hand grip strength (kg) | **-** | 29.8±10.9 | 32.3±12.2 | 0.08 |
| Phase angle (°) | **-** | 5.86±1.02 | 6.01±0.93 | 0.24 |
| Over-hydration(L) | **-** | 0.12±1.59 | 0.26±1.61 | 0.48 |
| Appendicular lean mass (kg) | **-** | 18.19±4.84 | 18.62±5.50 | 0.51 |
| Lean mass (kg) | **-** | 40.47±9.35 | 40.82±10.84 | 0.78 |
| Trunk fat mass (kg) | **-** | 12.67±5.09 | 12.22±4.76 | 0.49 |
| Fat mass (kg) | **-** | 23.44±8.74 | 22.38±7.75 | 0.33 |
| Appendicular lean mass index (kg/m^2^) | **-** | 6.71±1.32 | 6.93±1.51 | 0.23 |
| Lean mass index (kg/m^2^) | **-** | 14.99±2.49 | 15.26±3.01 | 0.44 |
| Fat mass index (kg/m^2^) | **-** | 8.87±3.57 | 8.53±3.02 | 0.44 |
| Group 1: patients that were evaluated only in the first assessment. Group 2: patients that were evaluated in the first and second assessment; p: unpaired Student t-test between Group 1 and Group 2. Data from the first assessment. ALM, appendicular lean mass; ALMI, appendicular lean mass index; BMI, body mass index; eGFR, estimated glomerular filtration rate; FM, fat mass; FMI, fat mass index; HGS, hand grip strength; LM, lean mass; LMI, lean mass index; OH, overhydration; PhA, phase angle; TrFM, trunk fat mass. ALM, ALMI, FM, FMI and TrFM data by dual energy X-ray absorptiometry analysis. PhA and OH data by bioelectrical impedance analysis. | | | | |
