# Supplementary material for: Bed-side measures for diagnosis of low muscle mass, sarcopenia, obesity, and sarcopenic obesity in patients with chronic kidney disease under non-dialysis-dependent, dialysis dependent and kidney transplant therapy
Source: PLoS One. 2020 Nov 20;15(11):e0242671. doi: 10.1371/journal.pone.0242671 (PMC7679152; doi:10.1371/journal.pone.0242671)
Supplement: S3 Table — (DOCX) [file pone.0242671.s008.docx]

| **S3 TABLE. Correlation between dual energy X-ray absorptiometry and bed-side measurements of muscle mass and adiposity** | | | | | |
| --- | --- | --- | --- | --- | --- |
| **Appendicular lean mass (kg) by dual energy X-ray absorptiometry analyze** | | | | | |
| **Bed-side Measurement** | **Female** | **Male** | **Bed-side Measurement** | **Female** | **Male** |
| Mid-arm muscle circumference (cm) | 0.48* | 0.59* | Fat free mass by body composition monitor (kg) | 0.67* | 0.65* |
| Arm muscle area (cm^2^) | 0.46* | 0.60* | Fat free mass by body composition monitor (%) | -0.19* | -0.23* |
| Corrected arm muscle area (cm^2^) | 0.46* | 0.60* | Fat free mass index by body composition monitor (kg/m^2^) | 0.51* | 0.39* |
| Adductor pollicis muscle thickness (mm) | 0.51* | 0.58* | Predicted fat free mass (kg) | 0.90* | 0.87* |
| Calf circumference (cm) | 0.73* | 0.70* | Predicted fat free mass (%) | -0.42* | -0.03 |
| Phase angle (°) | 0.20* | 0.08 | Predicted fat free mass index (kg/m^2^) | 0.63* | 0.65* |
| Body cell mass (kg) | 0.64* | 0.60* | Appendicular fat free mass (kg) | 0.90* | 0.90* |
| Body cell mass index (kg/m^2^) | 0.49* | 0.37* |  |  |  |
| **Fat mass index (kg/m^2^) by dual energy X-ray absorptiometry analyze** | | | | | |
| Bed-side Measurement | Female | Male | Bed-side Measurement | Female | Male |
| Weight (kg) | 0.80* | 0.76* | Conicity index | 0.38* | 0.52* |
| Body mass index (kg/m^2^) | 0.92* | 0.88* | Fat mass body composition monitor (kg) | 0.90* | 0.88* |
| Mid-arm circumference (cm) | 0.86* | 0.78* | Fat mass body composition monitor (%) | 0.79* | 0.82* |
| Waist circumference (cm) | 0.84* | 0.85* | Fat mass index body composition monitor (kg/m^2^) | 0.93* | 0.91* |
| Waist circumference for high ratio | 0.86* | 0.86* | pFM (kg) | 0.91* | 0.89* |
| Triceps skin fold thickness (mm) | 0.78* | 0.79* | pFM (%) | 0.89* | 0.85* |
| A body shape index | -0.20* | 0.06 | pFMI (kg/m^2^) | 0.96* | 0.93* |
| *Pearson correlation analysis between dual energy X-ray absorptiometry and be-side measurements, p≤0.05. AFFM, appendicular fat free mass; BCM, body cell mass; BCMI, body cell mass index; FFMBCM, fat free mass by body composition monitor; FFMIBCM, fat free mass index by body composition monitor; FMBCM, fat mass body composition monitor; FMIBCM, fat mass index body composition monitor; pFFM, predicted fat free mass; pFFMI, predicted fat free mass index; pFM, predicted fat mass; pFMI, predicted fat mass index; PhA, phase angle. PhA, BCM, BCMI, FFMBCM, FFMIBCM, FMBCM, FMIBCM, pFFM, pFFMI, pFM, pFMI and AFFM measures by bioelectrical impedance. AFFM by Sergi equation [20] and pFFM and pFM by Bellafronte equation [21]. FMBCM and FFMBCM by bioelectrical impedance from body composition monitor (Fresenius Medical Care). | | | | | |
