# Supplementary material for: Bed-side measures for diagnosis of low muscle mass, sarcopenia, obesity, and sarcopenic obesity in patients with chronic kidney disease under non-dialysis-dependent, dialysis dependent and kidney transplant therapy
Source: PLoS One. 2020 Nov 20;15(11):e0242671. doi: 10.1371/journal.pone.0242671 (PMC7679152; doi:10.1371/journal.pone.0242671)
Supplement: S4 Table — (DOCX) [file pone.0242671.s009.docx]

| **TABLE S4. Sensitivity and specificity of bed-side measures/indexes to identify low muscle mass, obesity, sarcopenia and sarcopenic obesity in CKD subgroups** | | | |
| --- | --- | --- | --- |
| **Variables** | **Sex** | **Sensitivity, % (95%CI)** | **Specificity, % (95%CI)** |
| **Low Muscle Mass^1^** | | | |
| AFFM (kg)  Men ≤ 21.43  Women ≤ 15.87 | *NDD* | 1.00 (0.82; 1.00) | 0.95 (0.85; 0.98) |
|  | *HD* | 0.90 (0.79 to 0.96) | 0.70 (0.48; 0.86) |
|  | *PD* | 0.91 (0.59; 0.99) | 1.00 (0.67; 1.00) |
|  | *KTx* | 0.92 (0.79; 0.98) | 0.84 (0.68; 0.93) |
| CC (cm)  Men ≤ 37.0  Women ≤ 35.5 | *NDD* | 0.91 (0.70; 0.98) | 0.88 (0.76; 0.94) |
|  | *HD* | 0.80 (0.66; 0.89) | 0.58 (0.36; 0.77) |
|  | *PD* | 0.83 (0.50; 0.97) | 0.81 (0.47; 0.96) |
|  | *KTx* | 0.76 (0.60; 0.87) | 0.71 (0.54; 0.84) |
| **Obesity^2^** | | | |
| FMIBCM (kg/m^2^)  Men > 8.82  Women > 12.58 | *NDD* | 1.00 (0.85; 1.00) | 0.87 (0.74; 0.94) |
|  | *HD* | 0.92 (0.62; 0.99) | 0.87 (0.76; 0.94) |
|  | *PD* | 1.00 (0.50; 1.00) | 0.95 (0.75; 0.99) |
|  | *KTx* | 0.90 (0.68; 0.98) | 0.96 (0.87; 0.99) |
| WC/H  Men > 0.60  Women > 0.66 | *NDD* | 1.00 (0.85; 1.00) | 0.74 (0.60; 0.84) |
|  | *HD* | 0.76 (0.45; 0.93) | 0.84 (0.73; 0.92) |
|  | *PD* | 1.00 (0.50; 1.00) | 0.90 (0.69; 0.98) |
|  | *KTx* | 0.95 (0.74; 0.99) | 0.83 (0.71; 0.91) |
| **Sarcopenia^3^** | | | |
| AFFM+HGS  Men AFFM ≤ 21.43kg + HGS < 27 kg  Women AFFM ≤ 15.87 kg + HGS < 16 kg | *NDD* | 1.00 (0.39; 1.00) | 0.98 (0.92; 0.99) |
|  | *HD* | 0.77 (0.40; 0.96) | 1.00 (0.91; 1.00) |
|  | *PD* | 1.00 (0.19; 1.00) | 1.00 (0.80; 1.00) |
|  | *KTx* | 1.00 (0.30; 1.00) | 1.00 (0.94; 1.00) |
| CC+HGS  Men CC≤ 37.0 cm + HGS < 27 kg  Women CC ≤ 35.5 cm _ HGS < 16 kg | *NDD* | 1.00 (0.39; 1.00) | 0.98 (0.92; 0.99) |
|  | *HD* | 0.77 (0.40; 0.96) | 1.00 (0.93; 1.00) |
|  | *PD* | 1.00 (0.19; 1.00) | 1.00 (0.80; 1.00) |
|  | *KTx* | 1.00 (0.30; 1.00) | 1.00 (0.94; 1.00) |
| **Sarcopenic Obesity^4^** | | | |
| FMIBCM+AFFM  Men FMIBCM > 8.82kg/m^2^ + AFFM ≤ 21.43kg  Women FMIBCM > 12.58 kg/m^2^ + AFFM ≤ 15.87 kg | *NDD* | 1.00 (0.30; 1.00) | 1.00 (0.94; 1.00) |
|  | *HD* | 0.62 (0.25; 0.89) | 0.92 (0.83; 0.97) |
|  | *PD* | - | - |
|  | *KTx* | 0.85 (0.42; 0.99) | 0.94 (0.86; 0.98) |
| ^1^Low Appendicular Lean Mass by Dual energy X-ray absorptiometry analysis, with <15kg for women and <20kg for men [10]. ^2^High Fat Mass Index by Dual energy X-ray absorptiometry analysis, with >13kg/m^2^ for women and >9kg/m^2^ for men [14]. ^3^Low Appendicular Lean Mass by Dual energy X-ray absorptiometry analysis in addition to low hand grip strength (<16kg for women and <27kg for men)[10].^4^ Fat Mass Index by Dual energy X-ray absorptiometry in addition to Low Appendicular Lean Mass by Dual energy X-ray absorptiometry. AFFM, appendicular fat free mass; CC, calf circumference; FMIBCM, fat mass index body composition monitor; HD, hemodialysis; KTx; kidney transplant; NDD, non dialysis-dependent; PD, peritoneal dialysis; WC/H, waist circumference for high ratio. AFFM and FMIBCM measures by bioelectrical impedance. AFFM by Sergi equation [20] and FMIBCM by bioelectrical impedance from body composition monitor (Fresenius Medical Care). It was not possible to evaluate sarcopenic obesity in PD group as there is no one diagnosed with. | | | |
