# Supplementary material for: Bed-side measures for diagnosis of low muscle mass, sarcopenia, obesity, and sarcopenic obesity in patients with chronic kidney disease under non-dialysis-dependent, dialysis dependent and kidney transplant therapy
Source: PLoS One. 2020 Nov 20;15(11):e0242671. doi: 10.1371/journal.pone.0242671 (PMC7679152; doi:10.1371/journal.pone.0242671)
Supplement: Attachment — Submitted filename: Review.docx [file pone.0242671.s010.docx]

To the editors, thank you for your consideration for evaluating the study. Here's the review:

- What are the main claims of the paper and how significant are they for the discipline?

This is a cross-sectional study (n 265) followed by a prospective study with 33% of the patients initially included (n 87).

The prospective information available on this topic in patients with chronic renal failure is scarce. In this sense, the paper provides relevant information comparing bed-sides measures with dual-energy X-ray absorptiometry in a cross-sectional study. It also tries to establish cut-off values for CKD patients.

- Are the claims properly placed in the context of the previous literature? Have the authors treated the literature fairly?

Yes

- Do the data and analyses fully support the claims? If not, what other evidence is required?

This is a cross-sectional study (n 265), followed by a prospective follow-up study with 33% (n 87) of the patients initially included. The study provides a detailed description of the prevalence of obesity, sarcopenia, and sarcopenic obesity in a cohort of patients with CKD (CKD Stages 1 to 3a, PD, hemodialysis, and kidney transplantation). The paper provides relevant information comparing bed-sides measures with dual-energy X-ray absorptiometry in a cross-sectional study in CKD patients. It also tries to establish cut-off values for CKD patients. However, the diagnostic capabilities of the measurements of muscle and fat mass are analyzed mainly according to sex and without considering the different situations of renal function; this makes it difficult to assess the sensitivity and specificity of the measures in different "renal situations" and also questions the validity of the cut-off points. An analysis of the diagnostic capacity of the tests in relation to the renal situation is necessary.

In the prospective study (n 87), the renal situation of the patients is not clear. A graph comparing the initial situation and follow-up according to the initial classification (renal and sarcopenic-obesity) would also be interesting.

The study excluded patients over 60 years of age, a group that constitutes an important and growing population with a high prevalence of CKD, sarcopenia, and obesity. This point should be discussed more extensively in the discussion.

Minor review:

Abstract: the acronyms DXA and HGS must be defined in the abstract on its first use.

It would be interesting to include a table of the different measures according to the renal situation.

Page 6, lines 6 and 7, the sentence is understood, but it isn't very clear.

- *PLOS ONE* encourages authors to publish detailed protocols and algorithms as supporting information online. Do any particular methods used in the manuscript warrant such treatment? If a protocol is already provided, for example for a randomized controlled trial, are there any important deviations from it? If so, have the authors explained adequately why the deviations occurred?

*Not applicable*

- If the paper is considered unsuitable for publication in its present form, does the study itself show sufficient potential that the authors should be encouraged to resubmit a revised version?

Yes

- Are original data deposited in appropriate repositories and accession/version numbers provided for genes, proteins, mutants, diseases, etc.?

Not applicable

- Are details of the methodology sufficient to allow the experiments to be reproduced?

Yes

- Is the manuscript well organized and written clearly enough to be accessible to non-specialists?

Yes
